# NEMO-Binding Domain Peptide Attenuates Lipopolysaccharide-Induced Acute Lung Injury by Inhibiting the NF-*κ*B Signaling Pathway

**DOI:** 10.1155/2016/7349603

**Published:** 2016-11-09

**Authors:** Jianhua Huang, Li Li, Weifeng Yuan, Linxin Zheng, Zhenhui Guo, Wenjie Huang

**Affiliations:** ^1^Department of Respiratory Medicine, General Hospital of Guangzhou Military Command of PLA, Guangzhou, Guangdong, China; ^2^Department of Pulmonary Medicine, Chenzhou No. 1 People's Hospital, Chenzhou, Hunan, China; ^3^Department of Medical Intensive Care Unit, General Hospital of Guangzhou Military Command of PLA, Guangzhou, Guangdong, China; ^4^Guangdong Provincial Key Laboratory of Geriatric Infection and Organ Function Support, Guangzhou, Guangdong, China

## Abstract

The aim of the present study is to investigate the protective effects and relevant mechanisms exerted by NEMO-binding domain peptide (NBD) against lipopolysaccharide- (LPS-) induced acute lung injury (ALI) in mice. The ALI model was induced by intratracheally administered atomized LPS (5 mg/kg) to BABL/c mice. Half an hour before LPS administration, we treated the mice with increasing concentrations of intratracheally administered NBD or saline aerosol. Two hours after LPS administration, each group of mice was sacrificed. We observed that NBD pretreatment significantly attenuated LPS-induced lung histopathological injury in a dose-dependent manner. Western blotting established that NBD pretreatment obviously attenuated LPS-induced I*κ*B-*α* and NF-*κ*Bp65 activation and NOX1, NOX2, and NOX4 overexpression. Furthermore, NBD pretreatment increased SOD and T-AOC activity and decreased MDA levels in lung tissue. In addition, NBD also inhibited TNF-*α* and IL-1*β* secretion in BALF after LPS challenge. In conclusion, NBD protects against LPS-induced ALI in mice.

## 1. Introduction

Acute lung injury (ALI) is caused by microbial infection, sepsis, trauma, and ischemia and reperfusion, leading to epithelial integrity disruption, neutrophil accumulation, noncardiogenic pulmonary edema, severe hypoxemia, and intense pulmonary inflammatory responses. The acute respiratory distress syndrome (ARDS) is a more severe form of ALI. Both ALI and ARDS are major causes of acute respiratory failure and leading causes of morbidity and mortality in critically ill patients [1, 2]. In recent years, rapid advances in supportive care, such as mechanical ventilation, have been achieved. However, several data analyses have shown that the mortality rate associated with ALI- or ARDS-induced acute respiratory failure is still high at approximately 40% [3–5]. The pathogenesis of ALI/ARDS is characterized by polymorphonuclear cells (PMNs) infiltration into the lungs, which may cause interstitial edema. In addition, the alveoli develop fibrin leakage, resulting in increases in the levels of macrophage-derived cytokines, chemokines, and other proinflammatory mediators in the lungs [6]. The results of previous studies indicate that many specific therapies have not proven beneficial with respect to managing ALI/ARDS [7]. Therefore, investigating the mechanisms underlying ALI/ARDS is necessary, as such investigations may contribute to the development of novel effective treatments for ALI/ARDS.

ALI research relies mainly on animal models. The intratracheal lipopolysaccharide (LPS) administration model is the most commonly used clinically relevant severe lung injury model for studying the pathophysiologic mechanisms underlying ALI, as it simulates the human disease [8]. LPS are components of gram-negative bacterial walls and play an important role in ALI by inducing PMNs infiltration into injured lung tissue, mimicking clinical ALI progression. TNF-*α* and keratinocyte-derived chemokines are secreted during this process and recruit intravascular PMNs into the alveolar spaces [9]. These activated PMNs generate superoxide anions (O_2_^−^) and release proteases via respiratory bursts and degranulation [10]. This excessive inflammatory response induces significant lipid peroxidation and antioxidant enzyme activity alterations, thereby disrupting lung endothelial integrity [11].

It is accepted that NF-*κ*B, a critical transcriptional factor, plays an important role in the pathogenesis of ALI/ARDS [12]. A variety of experimental techniques have demonstrated that NF-*κ*B exists in both the cytoplasm and the nucleus. NF-*κ*B activation induces its translocation from the cytoplasm to the nucleus. NF-*κ*B is activated by LPS and some cytokines, such as TNF-*α* and IL-1*β*. These cytokines initiate a cascade of events leading to I*κ*B phosphorylation by I*κ*B kinase (IKK), which triggers I*κ*B degradation by the ubiquitin–proteasome pathway. I*κ*B, an inhibitory protein, binds to P65 and P50, two NF-*κ*B subunits, under normal conditions. I*κ*B degradation removes a nuclear localization signal from NF-*κ*B, resulting in its uncoiling and translocation into the nucleus. This uncoiling is thought to activate the transcription of cytokines and other proinflammatory mediators [13, 14]. IKK comprises three subunits, IKK*α*, IKK*β*, and IKK*γ*, which are also collectively known as NEMO (NF-*κ*B essential modulator). IKK*γ* has no catalytic domain and plays a critical role in biology only when being a part of the IKK complex [15]. The NH2-terminus of NEMO associates with a hexapeptide sequence (Leu-Asp-Trp-Ser-Trp-Leu) within the COOH terminus of IKK*α* and IKK*β* termed the NEMO-binding domain (NBD). Previous studies have shown that LPS induces the NF-*κ*B activation required for NBD activity. NBD disrupts the association between NEMO and IKK*β* and blocks LPS-induced NF-*κ*B activation in cells, which ameliorates the inflammatory response and oxidative stress in distinct animal models to some extent [16, 17]. The results of previous studies indicate that understanding the mechanisms underlying the protective effects of NBD may facilitate the development of therapies that are effective against ALI.

Therefore, the aim of the current study was to elucidate the mechanisms underlying the protective effects exerted by NBD against LPS-induced ALI.

## 2. Materials and Methods

### 2.1. Chemicals and Reagents

LPS (from* Escherichia coli* 055: B5) was purchased from Sigma-Aldrich, St. Louis, MO, USA. NBD and N-NBD (negative control) were obtained from MERCK (NBD amino acid sequence: H-Asp-Arg-Gln-Ile-Lys-IIe-Trp-Phe-Gln-Asn-Arg-Arg-Met-Lys-Trp-Lys-Lys-Thr-Ala-Leu-Asp-Trp-Ser-Trp-Leu-Gln-Thr-Glu-OH; N-NBD amino acid sequence: H2N-Asp-Arg-Gln-Ile-Lys-IIe-Trp-Phe-Gln-Asn-Arg-Arg-Met-Lys-Trp-Lys-Lys-Thr-Ala-Leu-Asp-Ala-Ser-Ala-Leu-Gln-Thr-Glu-OH). Rabbit polyclonal antibodies against p-IKK*α*/*β*, IKK*α*, IKK*β*, p-I-*κ*B, I-*κ*B, p-NF-*κ*B p65, NF-*κ*B p65, NOX1, NOX2, and NOX4 were purchased from Cell Signaling Technology (Santa Cruz Biotechnology, Inc., Texas, USA). All secondary antibodies and *β*-actin were obtained from Boster (Wuhan Boster Bio-Engineering Limited Company, Wuhan, China). The TNF-*α*, IL-1*β*, IL-6, and IL-8 ELISA kits were obtained from Boster (Wuhan Boster Bio-Engineering Limited Company, Wuhan, China). The superoxide dismutase (SOD), total antioxidant capacity (T-AOC), and malondialdehyde (MDA) assay kits and the BCA Protein Assay Kit were obtained from Beyotime Biotech (Beyotime Biotech, Jiangsu, China).

### 2.2. Animals

Male BLAB/c mice weighing 18–22 g were obtained from the Laboratory Animal Center of Guangdong province (Guangdong, China). All mice were fed a normal standard diet and tap water ad libitum and were housed in an animal facility under controlled environmental and temperature (24 ± 1°C) conditions, 12 h light/dark cycles, and controlled humidity. All mice were allowed 7 days to adapt to their environments before the experiments. This study was approved by the Institutional Animal Ethics Committee of the General Hospital of Guangzhou Military Command of PLA and was carried out in compliance with the criteria outlined in the Provisions and General Recommendation of the Chinese Experimental Animals Administration Legislation.

### 2.3. LPS-Induced ALI Experimental Protocol

The animals were randomly assigned to one of six groups (*n* = 6 per group). Two control groups were intratracheally given atomized LPS (model group) or saline (control group). Three groups were experimental groups (NBD-2, NBD-6, and NBD-10 groups) that received intratracheal NBD at concentrations of 2, 6, and 10 *μ*g/50 *μ*L/mouse (small, middle, and large NBD groups, resp.) 30 min before intratracheal LPS (100 *μ*g/50 *μ*L/mouse) administration. The remaining group (N-NBD group), which served as a negative control, received a nonfunctional NBD analogue at a concentration of 6 *μ*g/50 *μ*L/mouse 30 min before intratracheal LPS (100 *μ*g/50 *μ*L/mouse) administration.

Two hours after the mice were given LPS or saline, they were sacrificed using sodium pentobarbital. Their right lung tissues were collected for histopathological and immunohistochemical analyses. Their left lungs were snap-frozen in liquid nitrogen and stored at −80°C for enzyme-linked immunosorbent assay (ELISA) and Western blotting analysis. Bronchoalveolar lavage fluid (BALF) samples were collected for protein and cell counting detecting.

### 2.4. Lung Histopathological Studies

After the mice were euthanized and subjected to thoracotomy, their right lungs were removed and tied off at the end of trachea to keep the lungs inflated. Then, the lungs were fixed in 4% paraformaldehyde for 18 h before being embedded in paraffin and sliced into 3 *μ*m thick sections using a microtome and stained with hematoxylin and eosin (H&E). Lung histologic changes were evaluated by pathologists who were blinded to the study. Lung parenchyma histological alterations were quantitatively graded using the following scale ranging from 0 to 5 [18]: (0) no reaction in the alveolar walls; (1) diffuse reaction in the alveolar walls—primarily neutrophilic—without alveolar wall thickening; (2) diffuse inflammatory cell (neutrophil and mononuclear cell) infiltration in the alveolar walls, with slight thickening; (3) distinct (2-3 times) alveolar wall thickening due to the presence of inflammatory cells; (4) alveolar wall thickening up to 25% above baseline; and (5) alveolar wall thickening up to 50% above baseline. The final score was calculated as the mean of scores from 50 microscopic fields.

### 2.5. Cytokine, Protein, and Cell Count Analyses

Blood samples were centrifuged at 4°C at 2,500 rpm for 15 min and used to estimate serum TNF-*α*, IL-1*β*, IL-6, and IL-8 levels. After thoracotomy, the trachea was identified and intubated with a tracheal cannula. The trachea and pulmonary alveoli were washed 3 times with 1 mL of sterile saline, and all lavage fluid was collected according to the study groups. The BALF was centrifuged at 500 ×g for 5 min at 4°C to obtain the supernatants, which were stored at −20°C for protein and cell counts assays. BALF protein concentrations were measured using a BCA Protein Assay Kit. Total cell counts were determined using a hemocytometer. The numbers of neutrophils were determined on BALF smear slides stained with Diff-Quick.

### 2.6. Measurement of SOD, T-AOC, and MDA Activity

SOD and T-AOC activity were measured using commercially available assay kits, according to the manufacturer's instructions. MDA levels were measured using a thiobarbituric acid reactive substances assay kit, according to the manufacturer's instructions.

### 2.7. Lung Tissue Western Blot Analysis

Frozen lung tissue samples were thawed and homogenized in radio-immunoprecipitation lysis buffer (RIPA) supplemented with protease inhibitors and phenylmethylsulfonyl fluoride (PMSF). After centrifugation, we measured protein concentrations via standard BCA assay. We subsequently added equal amounts of protein to 6x sodium dodecyl sulfate (SDS) loading buffer, after which the protein samples were heated (100°C; 5 min). The proteins were then separated by 10% SDS-PAGE and transferred to nitrocellulose membranes for the appropriate time. The membranes were blocked with Tris-buffered saline containing Tween-20 (TBST) and 5% nonfat milk (1 h; 24°C) and washed with TBS containing 0.1% Tween-20 before being incubated overnight at 4°C with the following antibodies: p-IKK*α*/*β*, IKK*α*, IKK*β*, p-I-*κ*B, I-*κ*B, p-NF-*κ*B p65, NF-*κ*B p65, NOX1, NOX2, and NOX4. The following day, the membranes were washed in TBST three times, incubated with a 1 : 2000 (v/v) dilution of horseradish peroxidase-labeled IgG for 1 h at 37°C, and then washed three additional times in TBST. The bands were visualized using ECL Plus Reagent, and the Western blot results were quantitated using Quantity One software (Gel-doc, Bio-Rad, Germany) and normalized to the *β*-actin signal. The blots were representative of multiple experiments.

### 2.8. Immunohistochemistry (IHC)

The lung tissue sections were incubated with 1 : 200 diluted mouse polyclonal antibodies against phospho-NF-*κ*Bp65 and NOX1 overnight at 4°C in a humidified chamber. Then, these immune complexes were incubated with the appropriate secondary antibody for 20 min at room temperature before being rinsed 3 times with PBS. Immunoreactivity was represented by brown staining using DAB (Beijing Biosynthesis Biotechnology Co., Ltd.). The sections were subsequently washed with distilled water, counterstained with H&E, and photographed via microscopy (Olympus Optical Co., Tokyo, Japan). The IHC staining results were evaluated by independent pathologists who were blinded to the study. Immunohistochemical staining intensity was scored using a scale ranging from 0 to 3 (negative = 0, weak = 1, moderate strong = 2, or strong = 3). Staining extent was assessed based on the percentages of positive cells as follows: 0 (negative), 1 (1–25%), 2 (26–50%), 3 (51–75%), and 4 (76–100%). The final staining score was calculated as the mean of the sums of the scores in five fields in every section, and all the sections were separated into low expression groups (final score = 1–5) and high expression groups (final score = 6–12).

### 2.9. Statistical Analysis

All data are expressed as the mean ± standard deviation (SD). Differences between the experimental and control groups were assessed by either the analysis of variance (ANOVA) or* t*-test, as applicable, using SPSS 18.0 (SPSS, 165 Inc.). Statistical significance was accepted at* P* < 0.05 for all analyses.

## 3. Results

### 3.1. Effect of NBD on Pulmonary Histopathological Changes in Mice with LPS-Induced ALI

**Figure 1:**
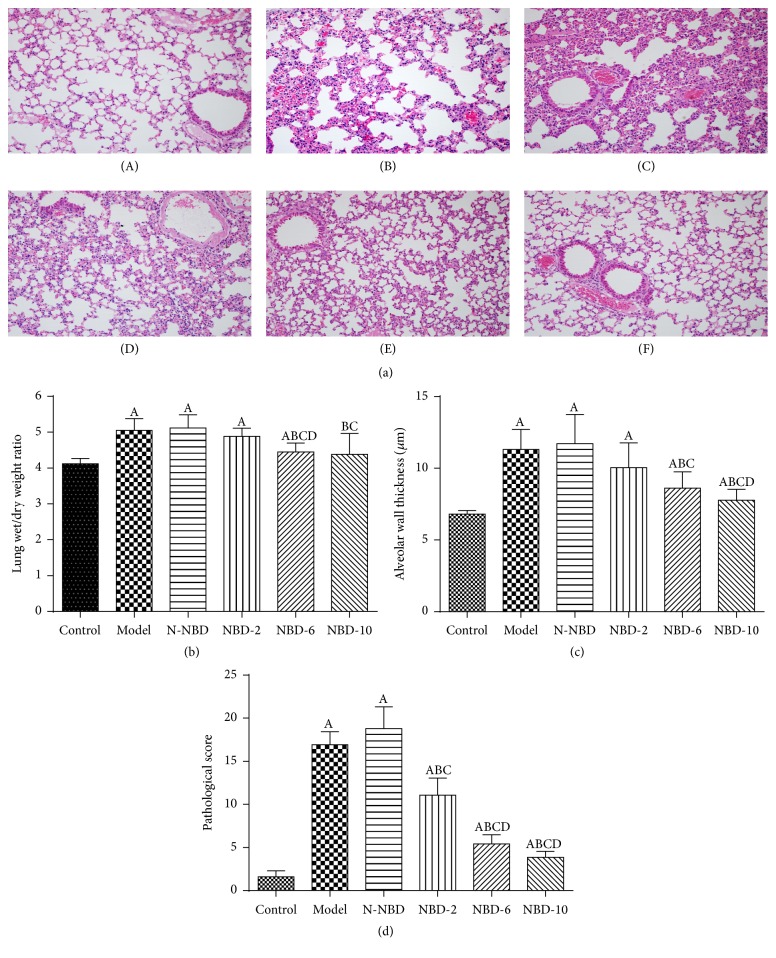
Effects of NBD on LPS-induced pulmonary histopathological changes in mice with ALI. (a) Lung sections stained with hematoxylin-eosin at 2 hours after LPS administration exhibited pulmonary histopathological changes (original magnification ×200). (A) Control group: normal structure. (B) Model group: alveolar wall thickening, hemorrhaging, alveolar collapse, and obvious inflammatory cell infiltration. (C) N-NBD group: same as the model group. (D) NBD-2 group. (E) NBD-6 group. (F) NBD-10 group. (b) Lung wet/dry weight ratios, (c) alveolar wall thickness, and (d) histopathological changes were evaluated to assess lung injury severity. LPS-induced lung injury severity was attenuated by NBD in a dose-dependent manner. Data are expressed as the mean ± SD (*n* = 6). A represents versus control group, ^A^ *P < *0.05; B represents versus model group, ^B^ *P*< 0.05; C represents versus N-NBD group, ^C^ *P < *0.05; D represents versus NBD-2 group, ^D^ *P < *0.05.

To evaluate the lung histopathological changes caused by LPS-induced lung injury, hematoxylin-eosin staining and histopathological analyses were performed. As expected, in the control group, normal pulmonary structures were observed via light microscopy, and no histopathological changes were noted. In the model group, staining revealed the presence of excessive edema and severe hemorrhage resulting in widespread increases in alveolar wall thickness, as well as alveolar collapse and obvious inflammatory cell infiltration. However, when the mice were treated with increasing doses of intratracheally administered NBD, the abovementioned LPS-induced pathological changes were attenuated (Figure 1(a)). Semiquantitative analysis of the NBD-treated lung tissue samples yielded similar results, as NBD treatment normalized the lung wet/dry weight ratio and attenuated the abovementioned increases in alveolar wall thickness and inflammation (Figures 1(b), 1(c), and 1(d)). These findings indicate that NBD pretreatment attenuates histopathological changes in lungs subjected to LPS-induced ALI.

### 3.2. Effects of NBD on BALF Protein Levels and Inflammatory Cell Infiltration

**Figure 2:**
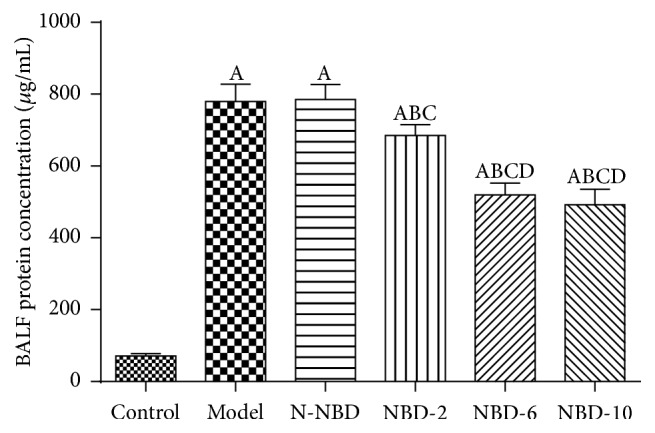
Effects of NBD on pulmonary vascular leakage in mice with ALI. BALF protein concentrations were assessed to evaluate pulmonary vascular leakage. The data indicated that NBD can noticeably reduce BALF protein levels. Data are expressed as the mean ± SD (*n *= 6). A represents versus control group, ^A^ *P < *0.05; B represents versus model group, ^B^ *P < *0.05; C represents versus N-NBD group, ^C^ *P < *0.05; D  represents versus NBD-2 group, ^D^ *P < *0.05.

**Figure 3:**
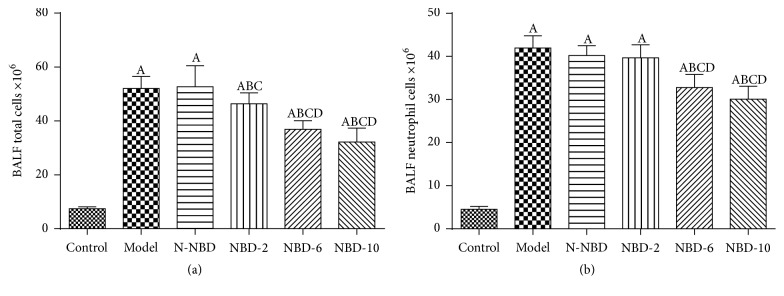
Effect of NBD on inflammatory cell infiltration in the BALF of mice with ALI. BALF (a) total cell and (b) neutrophil levels were assessed to evaluate inflammatory infiltration. Data are expressed as the mean ± SD (*n* = 6). A represents versus control group, ^A^ *P < *0.05; B represents versus model group, ^B^ *P < *0.05; C represents versus N-NBD group, ^C^ *P < *0.05; D represents versus NBD-2 group, ^D^ *P < *0.05.

Uncontrolled inflammation causes vascular leakage in the lung in CLP-induced acute lung injury. BALF protein levels were assessed at 2 h after LPS injection to evaluate the effects of NBD on alveolar-capillary membrane barrier integrity and pulmonary vascular leakage. The results indicated that the total protein concentration in the BALF was increased after LPS administration. However, the total protein concentration was markedly decreased in a dose-dependent manner in the NBD group compared with the ALI group (*P* < 0.05) (Figure 2). The total numbers of cells and neutrophils in the BALF were also quantified at 2 h after LPS administration. The results indicated that the total numbers of cells and neutrophils in the BALF were significantly increased in the ALI group compared to the control group (*P* < 0.05). Similar to the above results, NBD treatment significantly decreased the total numbers of cells and neutrophils in the BALF (*P* < 0.05) (Figure 3).

### 3.3. Effects of NBD on Cytokine Levels in Mice with ALI

TNF-*α* and IL-1*β* both are the major proinflammatory cytokines that facilitate active PMNs recruitment into the lungs in all kinds of pulmonary injury [10, 19, 20].

**Figure 4:**
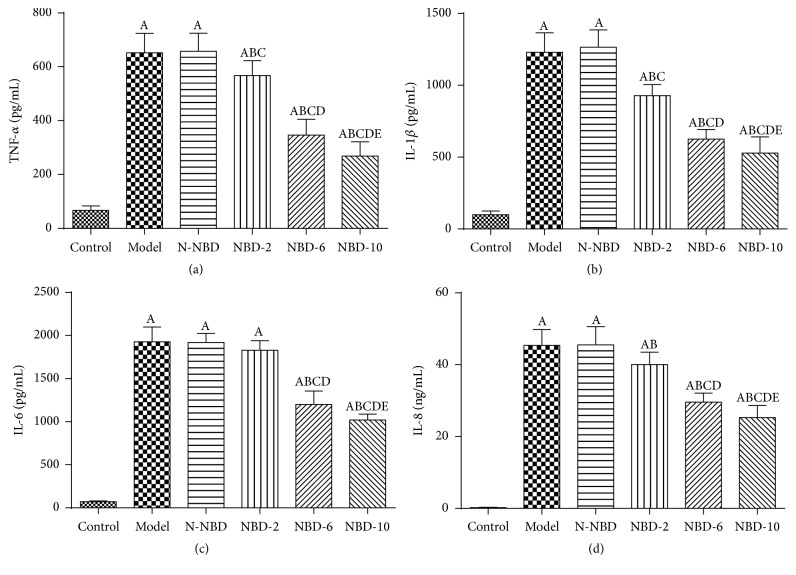
Effect of NBD on serum cytokine concentrations in mice with ALI. Values are expressed as the mean ± SD (*n* = 6). Increased serum TNF-*α*, IL-1*β*, IL-6, and IL-10 levels were detected in mice in response to LPS or N-NBD + LPS treatment. NBD decreased serum cytokine levels in ALI mice in a dose-dependent manner. A represents versus control group, ^A^ *P < *0.05; B represents versus model group, ^B^ *P < *0.05; C represents versus N-NBD group, ^C^ *P < *0.05; D represents versus NBD-2 group, ^D^ *P < *0.05; E represents versus NBD-6 group, ^E^ *P < *0.05.

At 2 hours after LPS administration, we observed that TNF-*α* and IL-1*β* expression levels had increased significantly in mice in the ALI group. However, mice in the NBD group exhibited concentration-dependent decreases in TNF-*α* and IL-1*β* levels (Figures 4(a) and 4(b)). IL-6 and IL-8 expression levels were also assessed to evaluate inflammation severity in ALI mice. The results showed that mice pretreated with NBD exhibited lower serum/BALF IL-6 levels after LPS challenge. Interestingly, NBD exerted dose-dependent effects on the levels of these cytokines (Figures 4(c) and 4(d)). These data suggest that NBD pretreatment attenuates LPS-induced ALI by suppressing proinflammatory cytokine production.

### 3.4. Effect of NBD on SOD and T-AOC Activity and MDA Concentrations in the Lung Tissues of Mice with ALI

**Figure 5:**
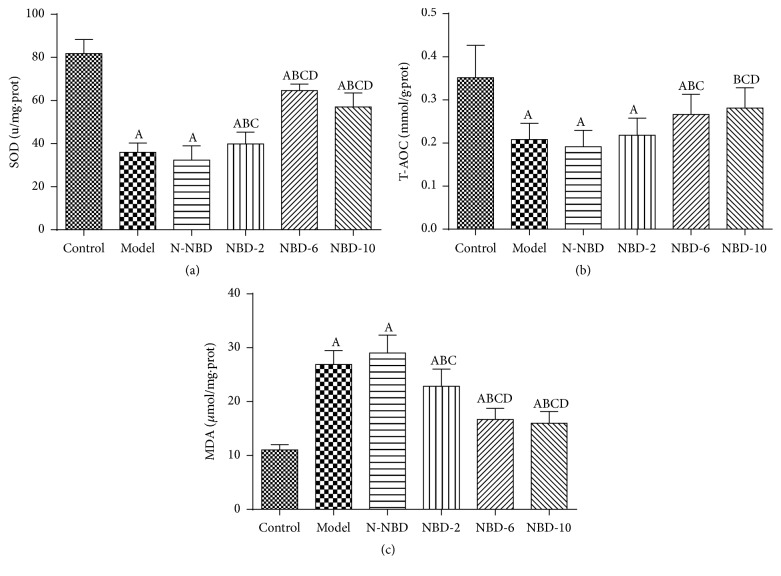
Effect of NBD on SOD and T-AOC activity and MDA concentrations in the lung tissues of mice with ALI. LPS challenge significantly increased MDA levels and decreased SOD and T-AOC activity compared with sham controls; however, treatment with the middle and large NBD concentrations exerted effects contrasting with those described above. Data are expressed as the mean ± SD (*n* = 6). A represents versus control group, ^A^ *P < *0.05; B represents versus model group, ^B^ *P < *0.05; C  represents versus N-NBD group, ^C^ *P < *0.05; D represents versus NBD-2 group, ^D^ *P < *0.05.

In general, the process of LPS-induced ALI is characterized by excessive oxidative stress, which damages endothelial barrier integrity [21]. SOD and T-AOC are antioxidant enzymes that are inactivated by reactions involving ROS and membrane phospholipids that form MDA. To assess the effects of NBD on MDA production in LPS-induced ALI, we detected SOD and T-AOC activity in the lung. LPS administration significantly decreased SOD levels (Figure 5(a)) and T-AOC (Figure 5(b)) activity and increased MDA (Figure 5(c)) levels. However, the middle and large NBD concentrations exerted effects contrasting with those of sham treatment. Therefore, we concluded that NBD attenuates ALI-induced oxidative stress in LPS-treated mice.

### 3.5. Effects of NBD on IKK, I*κ*Β, and NF-*κ*B p56 Activation in the Lung Tissues of Mice with ALI

**Figure 6:**
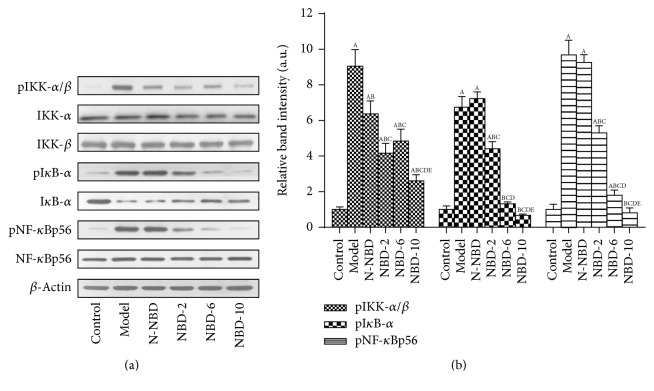
Effect of NBD on IKK, I*κ*Β-*α*, and NF-*κ*Bp65 activation in the lung tissues of mice with ALI. (a) Relative gray scale quantitation of total I*κ*B-*α*, NF-*κ*B, and *β*-actin phosphorylation. (b) Statistical results. IKK, I*κ*Β-*α*, and NF-*κ*Bp65 phosphorylation levels were increased by LPS administration, and these changes were inhibited by NBD pretreatment in a concentration-dependent manner. Data are expressed as the mean ± SD (*n* = 6). A represents versus control group, ^A^ *P* < 0.05; B represents versus model group, ^B^ *P* < 0.05; C represents versus N-NBD group, ^C^ *P* < 0.05; D represents versus NBD-2 group, ^D^ *P* < 0.05; E represents versus NBD-6 group, ^E^ *P* < 0.05.

**Figure 7:**
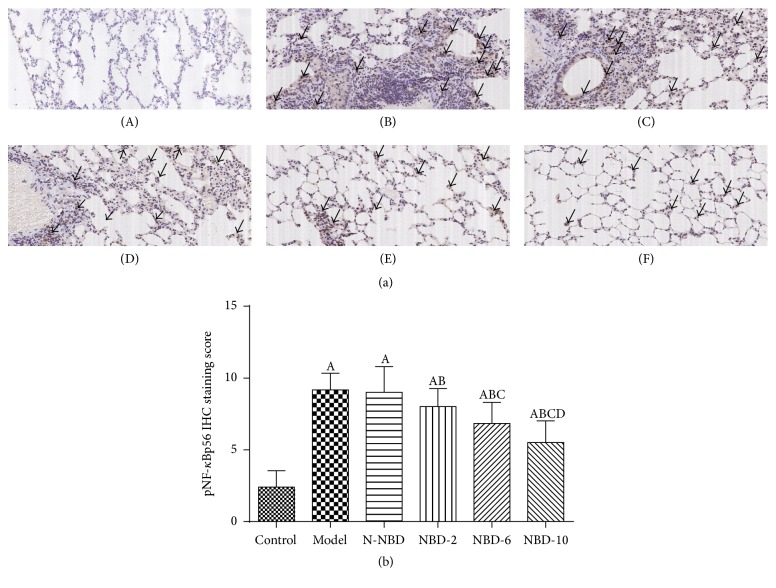
Effect of NBD on pNF-*κ*Bp65 expression, as determined via immunohistochemistry, in the lung tissues of mice with ALI. (a) Representative photographs of pNF-*κ*B P65 expression in the lung (original magnification ×200). (A) Control group; (B) model group; (C) N-NBD group; (D) NBD-2 group; (E) NBD-6 group; (F) NBD-10 group. Black arrows indicate the immunohistochemically positive pNF-*κ*Bp65. (b) IHC staining scores pertaining to pNF-*κ*B P65 expression. pNF-*κ*Bp65 expression levels were increased by LPS administration, and these changes were inhibited by NBD pretreatment in a concentration-dependent manner. Data are expressed as the mean ± SD (*n* = 6). A represents versus control group, ^A^ *P < *0.05; B represents versus model group, ^B^ *P < *0.05; C represents versus N-NBD group, ^C^ *P < *0.05; D represents versus NBD-2 group, ^D^ *P < *0.05.

It is accepted that LPS induces NF-*κ*B activation by acting on IKK and I*κ*Β phosphorylation in lung tissue and that this effect is related to the inflammatory response [21]. At 2 hours after LPS administration, IKK, I*κ*Β-*α*, and NF-*κ*Bp65 phosphorylation levels increased dramatically. However, these increases were significantly attenuated in a concentration-dependent manner in mice treated with NBD before LPS administration (Figures 6(a) and 6(b)). IHC staining and semiquantitative analysis yielded similar results (Figure 7).

### 3.6. Effects of NBD on NOX1, NOX2, and NOX4 Expression in the Lung Tissues of Mice with ALI

**Figure 8:**
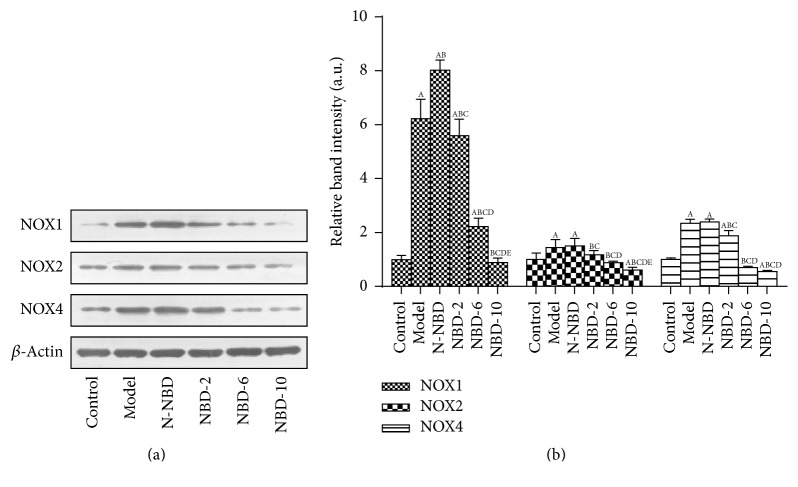
Effect of NBD on NOX1, NOX2, and NOX4 expression in the lung tissues of mice with ALI. (a) Relative NOX1, NOX2, NOX4, and *β*-actin immunointensity levels were calculated. (b) Statistical results. NOX1, NOX2, and NOX4 expression levels were increased by LPS administration, and these changes were inhibited by the middle and large NBD concentrations. Data are expressed as the mean ± SD (*n* = 6). A represents versus control group, ^A^ *P < *0.05; B represents versus model group, ^B^ *P < *0.05; C represents versus N-NBD group, ^C^ *P < *0.05; D represents versus NBD-2 group, ^D^ *P < *0.05; E represents versus NBD-6 group, ^E^ *P < *0.05.

**Figure 9:**
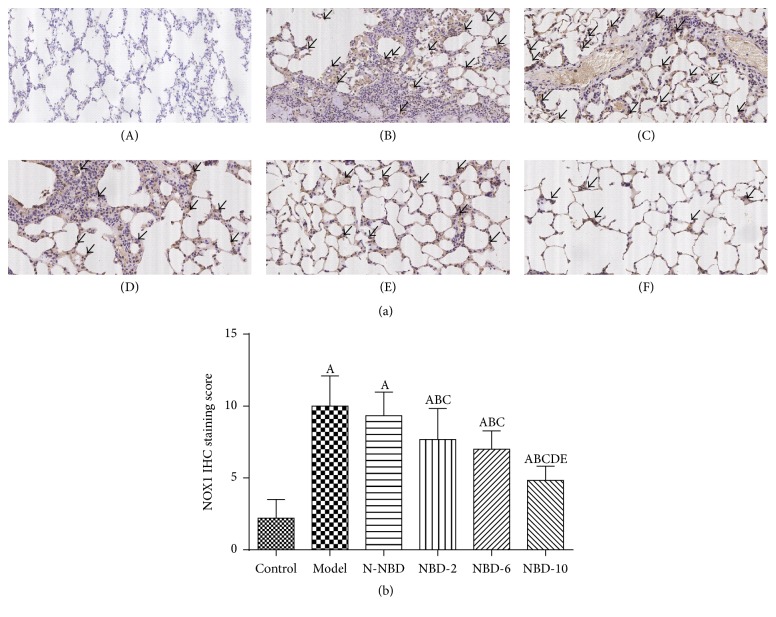
Effect of NBD on NOX1 expression, as determined via immunohistochemistry, in the lung tissues of mice with ALI. (a) Representative photographs of NOX1 expression in the lung (original magnification ×200). (A) Control group; (B) model group; (C) N-NBD group; (D) NBD-2 group; (E) NBD-6 group; (F) NBD-10 group. Black arrows indicate the immunohistochemically positive NOX1. (b) IHC staining scores pertaining to NOX1 expression. NOX1 expression levels were increased by LPS administration, and these changes were inhibited by NBD pretreatment in a concentration-dependent manner. Data are expressed as the mean ± SD (*n* = 6). A represents versus control group, ^A^ *P < *0.05; B represents versus model group, ^B^ *P < *0.05; C represents versus N-NBD group, ^C^ *P < *0.05; D represents versus NBD-2 group, ^D^ *P < *0.05; E represents versus NBD-6 group, ^E^ *P < *0.05.

Inflammation-induced oxidative stress plays an important role in ALI. Our previous results indicated that the NF-*κ*B signaling pathway regulates NOX family expression, thereby inducing increased reactive oxygen species production leading to cellular oxidative damage. Notably, LPS stimulation significantly increased NOX1, NOX2, and NOX4 expression, while pretreatment with the middle and large NBD concentrations suppressed LPS-induced NOX family member expression (Figures 8(a) and 8(b)). These results demonstrated that NBD attenuates LPS-induced oxidative stress in ALI by inhibiting NOX production. Similar results were observed via IHC staining and semiquantitative analysis (Figure 9).

## 4. Discussion

This study demonstrated the effects exerted by NBD on LPS-induced inflammation and oxidative stress in mouse lung tissue. We found that NBD effectively prevented LPS-induced lung histopathological changes. We also observed that NBD pretreatment attenuated LPS-induced proinflammatory cytokine expression and oxidative stress and inhibited the NF-*κ*B pathway.

In experimental models of ALI, intratracheal LPS administration is the classic method of mimicking the process of human ARDS and causes similar histopathological changes, such as hemorrhage, interstitial edema, and neutrophil infiltration [22]. LPS also induces significant increases in pulmonary microvascular permeability, leading to increases in extravascular lung water, as well as protein-rich fluid leakage from the intravascular space into the interstitium and air spaces [23–25]. We observed similar histopathological changes in this study. Moreover, in this study, NBD pretreatment reversed the lung histopathological damage caused by LPS administration and attenuated protein leakage and activated PMN recruitment to the lung parenchyma. Thus, we speculated that the protective effects exerted by NBD are associated with NADPH oxidases, which contribute to the oxidative stress response in all types of inflammatory disorders. As we expected, NBD pretreatment reduced TNF-*α* and IL-1*β* release via NADPH oxidase suppression.

LPS activates alveolar macrophages and endothelial cells, increasing the levels of proinflammatory mediators, such as TNF-*α*, IL-6, and IL-8, which play major roles in inducing ALI by recruiting PMNs to the lungs and enhancing PMN activity [26]. IL-1*β* is another important proinflammatory cytokine that is generated mainly by macrophages and is involved in inflammatory and immunomodulatory processes [27]. IL-1*β* is a major extracellular proinflammatory cytokine that usually exerts synergistic effects with TNF-*α* [28]. Most studies have reported that TNF-*α* and IL-1*β* levels are increased in LPS-treated mice [29, 30]. These increases were suppressed by NBD in a concentration-dependent manner, demonstrating that NBD has significant anti-inflammatory properties, which may explain how NBD can inhibit cytokine production by inhibiting the NF-*κ*B signaling pathway. Previous studies showed that NF-*κ*B signaling pathway overactivation is closely related to ALI development [31, 32]. NF-*κ*B signaling is upregulated to induce the transcription of some inflammatory factors in LPS-induced ALI [12, 33]. Under resting conditions, NF-*κ*B is bound by I*κ*B and maintained in a deactivated state in the cytoplasm. LPS administration induces toll-like receptors to stimulate a series of NF-*κ*B cascades, resulting in I*κ*B depolymerization and degradation by proteasomes. The liberated NF-*κ*B translocates into the nucleus and binds to specific gene sequences, including gene promoter sequences or enhancer regions, suggesting that NBD-mediated NF-*κ*B inhibition may be caused by I*κ*B-*α* inhibition. This mechanism entails the binding of NBD to IKK*γ* to block IKK assembly, which reduces IKK activity and inhibits NF-*κ*B activation [34].

Inflammation-induced oxidative stress plays an important role in acute lung injury. The respiratory burst is an early symptom of LPS-induced acute lung injury that depends on PMNs producing reactive oxygen species (ROS), including O_2_^−^ and hydrogen peroxide. The respiratory burst can protect organs from pathogens. However, excessive ROS can damage tissues and cause inflammation [35]. ROS attack polyunsaturated fatty acids, producing lipid peroxidation products, such as MDA, causing tissue damage [36]. In our study, pretreatment with the middle and large NBD concentrations prevented MDA generation in the lung tissues of mice with LPS-induced ALI. ROS elimination requires antioxidant enzymes, such as SOD and T-AOC, which convert O_2_^−^ into H_2_O_2_, and catalase, which metabolizes H_2_O_2_ into hydrogen oxide and oxygen [37]. The present study revealed that LPS significantly reduced SOD and T-AOC activity in the lung. This finding is consistent with those pertaining to the effects exerted by inflammatory responses in ALI patients [37]. However, our study demonstrated that NBD treatment before LPS administration can reverse the decreases in SOD and T-AOC activity caused by LPS. Therefore, we hypothesized that NBD can reduce the inflammatory response by attenuating oxyradical formation in mice with LPS-induced ALI.

In conclusion, NBD treatment attenuates ALI by inhibiting LPS-induced NF-*κ*B signaling pathway overactivation, thereby suppressing LPS-induced inflammation and oxidative stress. Our findings suggest that NBD has potential as a therapeutic target in the treatment of acute inflammatory diseases, including ALI, ARDS, and infectious diseases.
